# Biphasic Drug Release from Rolled-Up Gelatin Capsules with a Cylindrical Cavity

**DOI:** 10.3390/pharmaceutics13122040

**Published:** 2021-11-29

**Authors:** Jihane Mzoughi, Thierry Vandamme, Valeriy Luchnikov

**Affiliations:** 1 Institut de Science des Matériaux de Mulhouse, CNRS, UMR 7361, Université de Haute-Alsace, F-68100 Mulhouse, France; jihane.mzoughi@uha.fr; 2 INSERM, Regenerative Nanomedicine UMR 1260, Centre de Recherche en Biomédecine de Strasbourg (CRBS), Université de Strasbourg, F-67000 Strasbourg, France; vandamme@unistra.fr

**Keywords:** biphasic drug release, chronotherapy, chronomodulated drug release, sustained release

## Abstract

Biphasic drug delivery systems are used for quick release of a specific amount of drug for immediate amelioration of a patient’s state, followed by sustained release, to avoid repeated administration. This type of delivery is often necessary for pain management and the treatment of many pathologies, such as migraines, hypertension, and insomnia. In this work, we propose a novel architecture of a biphasic release media that does not need the rapidly disintegrating layer and that allows for easily setting the sustained release rate. A drug-containing capsule is made by rolling up a thermally crosslinked gelatin strip on which drug reservoirs are formed by casting. The quick-release reservoir (QRR) is placed at the strip’s extremity, from which the rolling starts, while the sustained-release reservoir (SRR) is formed in the middle part of the strip. The strip is rolled around a cylinder that is a few millimeters wide, which is removed after rolling. The roll is stabilized by transglutaminase-catalyzed crosslinking of the consecutive shells. A biphasic release is successfully demonstrated with the use of model fluorescent drugs for single-dye and double-dye systems in phosphate-buffered saline (PBS) solution with pH = 7.4. In vitro, the drug from the QRR, placed at the walls of the cavity of the roll, is released immediately upon the capsule’s contact with the PBS solution. The drug from the SRR, embedded between the roll’s layers, diffuses steadily, with the lag time defined by the radial position of the reservoir.

## 1. Introduction

It is known that a well-scheduled drug release can significantly improve the efficiency of pharmacotherapies. In particular, a good therapeutic effect can be achieved by quick release (QR) of a significant amount of a drug, called a “loading-dose”, enabling the drug to rapidly reach the therapeutic level in blood plasma, followed by sustained release (SR) of the “maintenance-dose”, holding the therapeutic concentration level for a therapeutically sufficient period of time. This quick/slow biphasic drug treatment may be beneficial for pain management [1] and for treating the acute symptoms of diseases, such as migraines [2], insomnia [3] and hypertension [4]. Non-steroidal anti-inflammatory drugs (NSAIDs) are suitable candidate drugs for this type of administration [5]. A popular means for achieving a biphasic release is a bilayer tablet [6,7], usually prepared on the base of a microcrystalline cellulose excipient. One layer of the tablet provides QR of the drug due to the presence of a super disintegrant, such as croscarmellose sodium [8], while the second layer provides SR. Such tablets are simple and economical to fabricate by sequential compaction of loose powder layers, and they are gaining interest within the pharmaceutical industry. 

Recently, three dimensional (3D)-printing has become a popular approach for fabrication of bilayer tablets, offering additional possibilities for personalization of the dosage and release kinetics [9,10]. Moreover, the biphasic release effect was achieved with the use of alternative formulations, such as coaxially electrospun core/sheath nanofibers and polyvinylpyrrolidone and ethylcellulose serving office of the sheath polymer and the core matrix, respectively [11]. Chitosan/fucoidan nanoparticles were used to develop a nanoformulation for pulmonary delivery of gentamicin exhibiting a biphasic release characteristic [12]. In all of these two-phase release systems, more than one polymer is used and different additives have been incorporated in order to adjust certain properties; relatively complex methods have also been applied. The components of each layer must be carefully chosen. Thus, it is challenging to find a concept that is easy to implement based on a single polymer.

In this paper, we propose a new design for biphasic release media. The biphasic release effect is achieved due to the specific geometry of the capsules, rather than due to their chemical composition. Within this approach, the capsules are formed by rolling up a (bio)polymer strip, for instance a crosslinked gelatin strip, around a cylindrical support, which is then removed. Prior to rolling, drug reservoirs are formed on the surface of the strip in such a manner that the reservoir containing the QR dose is located on the inner side of the obtained hollow-core cylinder, while the reservoir accommodating the SR dose is buried between consecutive shells of the roll. Because the QR reservoir (QRR) is placed on the hidden surface of the roll, there is no risk of a patient or doctor accidentally touching the drug or the reservoir being damaged by contact with other capsules in a container. The drug contained in the reservoir is dissolved immediately upon penetration of the physiological media, such as the intestinal fluid, in the cavity. The SR is swelling-controlled and diffusion-controlled and the lag time is determined by the radial position of the SR reservoir (SRR) inside the cylindrical wall of the capsule. 

The application of rolled-up delivery systems for pharmaceutical formulations has been known since 1971, when Higuchi patented a drug-delivery device made by rolling an impermeable film, soluble in body fluids and covered by a drug [13]. In his invention, degradation of the film, curled around itself in the spiral-like manner, was claimed to enable arbitrary release kinetics due to the possibility of distributing the drugs in a complex manner along the spiral. However, to the best of our knowledge, this device did not find broad acceptance, probably because it was limited to the case of the erosion-controlled release, which seems to not be well suited for realizing well-defined reproducible kinetics due to the complexity and irregularity of the erosion process. Recently, we explored the rolling-up approach for programmed release, controlled either by longitudinal swelling of a chitosan fiber, embedded in a hydrophobic polydimethylsiloxane matrix [14], or by diffusion in the radial direction across the consecutive layers of the scroll of chitosan acetate film [15]. Therefore, the present work combines the previous findings on the swelling and diffusion-controlled SR from the rolled-up capsules with the idea of using the central cavity in the capsule for the QR.

## 2. Materials and Methods

### 2.1. Preparation of Crosslinked Gelatin Films

The gelatin film forming solution was prepared by dissolving 5 wt.% of bovine gelatin powder (Sigma-Aldrich, Taufkirchen, Germany) in distilled water at 60 °C for 30 min. Then, 60-µm thick films were obtained by casting the gelatin film forming solution over a covered Mylar® (DuPont Teijin Films^TM^, Cotern, Luxembourg) mold. The evaporation of water was carried out at 22 ± 2 °C and at 50 ± 10% relative humidity. Dehydrothermal (DHT) crosslinking [16] of the gelatin films was achieved by drying the films at 100 °C for 1 h, then heating them to 150 °C for 8 h in a Memmert UFE500 oven (Memmert GmbH, Schwabach, Germany). This DHT treatment time was chosen as the compromise between the fabrication convenience and the achieved performance properties of the films (see Appendix A). The treatment formed crosslinks through a condensation reaction between the carboxyl and amino groups in the gelatin. The samples were stored for 1–2 days at room temperature in a vacuum desiccator before being cut into 20 cm long strips using an infrared laser machine (Laser solution LS100, Gravotech Marking, La Chapelle Saint-Luc, France) and making the capsules.

### 2.2. Crosslinking Extent Determination Using the 2,4,6-Trinitrobenzene Sulfonic Acid (TNBSa) Method

The extent of crosslinking was determined by measuring the amount of free or unreacted amino groups in each gelatin film [17]. The measurement protocol was adapted from a procedure described by Kale and Bajaj [18] (cf. Appendix B). The degree of crosslinking was estimated as 13 crosslinks per molecule of 1000 amino acid residues. 

### 2.3. Loading the Gelatin Strips Using Fluorescent Probes

The model drug reservoirs were formed by spreading solutions of fluorescent probes (Fluorescein Disodium (FD) (Alfa Aesar™, Ward Hill, MA, USA) or Rhodamine B (RhB) (Sigma Aldrich, Taufkirchen, Germany) over the surface of a gelatin stripe at areas corresponding to the prescribed radial positions of the reservoirs inside the capsule after the rolling. Next, 20 μL of a solution per reservoir was spread using a micropipette. The concentration of the solution was 25 µg/µL, corresponding to a content of 500 µg within each reservoir. Reservoirs R_0_, R_1_, R_2_ and R_3_ were formed separately or in pairs on the gelatin stripe at precalculated distances so that each reservoir covered a full turn in the capsule (cf. Appendix C). R_0_ was located at the end of the stripe and it partially covered the internal surface of the capsule; therefore, it was in direct contact with the central cavity of the capsule after rolling. The R_1_, R_2_, and R_3_ reservoirs were formed in such a way so that after rolling they are located on the 3rd, 6th and 8.5th turn, respectively; the total number of full turns was equal to 10. The coordinates of the boundaries with respect to the end of the gelatin strip, from which the rolling started, are given in Table 1.

### 2.4. Fabrication of the Capsules Using the Rolling-Up Approach

**Figure 1:**
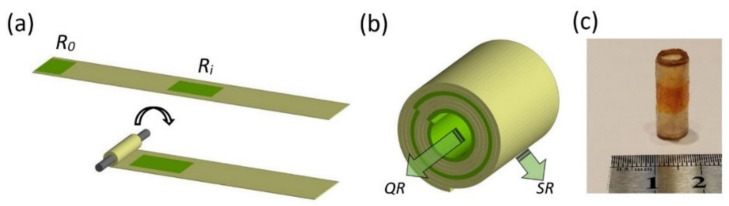
Formation of the capsules using the rolling-up approach. (**a**) Rolling of a gelatin strip with two drug reservoirs around a cylindrical stick. (**b**) Cross-section of the capsule with two reservoirs. The arrows symbolize the QR from the cavity of the capsule and the SR through the gelatin layers. (**c**) A photo of a rolled-up cylinder-like capsule.

The gelatin strips were rolled around a 5.5 mm diameter cylindrical stick, which was subsequently removed to form a cylinder-like capsule (Figure 1). To avoid the unrolling of the capsules, point-like drops of 20 wt.% Ajinomoto meat glue (Ajinomoto, Tokyo, Japan) were applied at the extremities of the film (away from the reservoirs) at each turn during the rolling. It is important to note that the Ajinomoto meat glue contained sodium caseinate E469, maltodextrin, transglutaminase and sunflower lecithin E322. Transglutaminase participated in the reaction that catalyzes the formation of the isopeptide bonds between the glutamine residue of γ-carboxamide and the primary ε-amine groups of gelatin [19].

### 2.5. Swelling Capacity of the Matrix

The swelling tests were conducted according to the Beaker test method. The capsule without the model drug reservoirs was weighed and placed inside a beaker. Then, 200 mL of phosphate-buffered saline (PBS) at pH = 7.4 was poured into the beaker at 37 °C. The swollen capsule was separated using a filter paper. By weighting the capsule, the swollen degree of the film was determined using the following formula:

(1) $$SD=\frac{W_{1}-W_{0}}{W_{0}}\times 100$$

where *W*_0_ is the weight of the sample before immersion in the buffer solution and *W*_1_ is the weight of the sample at time t. The swelling measurements were performed every 30 min for 8 h. The same protocol was applied to the swelling measurements in the buffer solutions at pH = 4.5 and at pH = 2, prepared according to the French Pharmacopeia [20].

### 2.6. In Vitro Release Study 

The dissolution testing experiments of the dosage forms were performed using the United States Pharmacopeia (USP) dissolution apparatus 2 (Agilent 708-DS, Agilent Technologies, Santa Clara, CA, USA) with a 50-rpm rotation speed at 37 °C. PBS was used as the dissolution medium. The pH of the PBS was adjusted to 7.4. All the experiments were performed in triplicate. FD and RhB have an ultraviolet (UV) emission peak at 510 nm and 540 nm, respectively. The amount of the released FD and RhB from the capsules was determined by fluorimetry (Thermo Scientific ™ Varioscan ™ LUX multimode microplate reader) using predetermined calibration curves for FD and RhB. The choice of FD and RhB was based on their emissions at distinct wavelengths, allowing us to avoid overlapping of the emission spectra.

## 3. Results

### 3.1. Swelling Behavior of the Capsules

**Figure 2:**
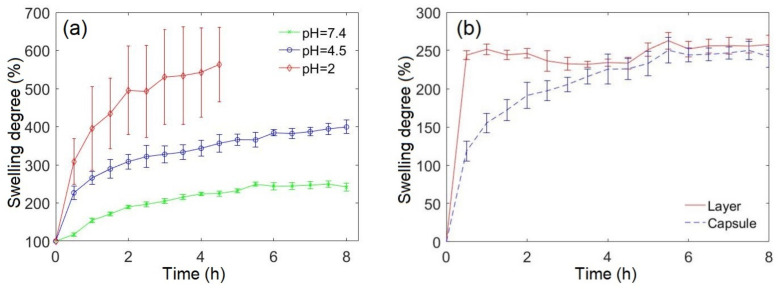
(**a**) Swelling behaviour of the capsules at pH = 2, pH = 4.5 and pH = 7.4. (**b**) Swelling kinetics of the capsules and the thermally crosslinked gelatin films in PBS (pH = 7.4).

Swelling of capsules was explored at pH = 2, approximately corresponding to stomach level, at pH = 4.5, and at pH = 7.4 (Figure 2a). In the most acidic medium (pH = 2), transglutaminase is deactivated [21] and did not perform the crosslinking of the roll’s layers to each other. As a consequence, the capsules unrolled after about 4 h with a gain in mass equal to approximately 550%. In a medium of pH = 4.5, the capsules showed a swelling capacity equal to approximately 400%, but remained rolled in the majority of the experiments. At pH = 7.4, the maximal weight uptake by the capsules was 250%, approximately, and the capsules demonstrated excellent resistance to unrolling.

Taking into account the stability issues of the capsules, we decided to concentrate this study on the swelling and the drug release at pH = 7.4, which corresponds approximately to the small intestine pH range, varying from pH = 6 in the duodenum to pH = 7.4 in the terminal ileum [22]. Figure 2b compares the swelling behavior of the capsules to that of the thermally crosslinked gelatin films. The crosslinked gelatin film allows water to diffuse easily into it and to reach approximately 250% of its initial mass after 30 min. The water uptake competes with the partial dissolution of the uncrosslinked gelatin. This results in a slight drop in the degree of swelling after 3 h. The capsules show a different swelling behavior, swelling to around 60% of the maximal swelling capacity within 1 h.

### 3.2. Monophasic and Biphasic Drug Release

**Figure 3:**
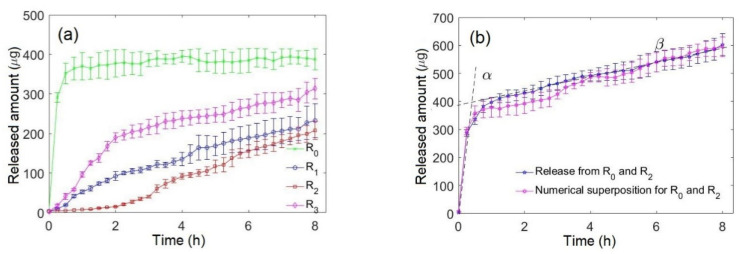
The kinetics of FD release from the rolled-up capsules. (**a**) Monophasic release from a single reservoir formed at different radial positions inside the capsules. (**b**) Biphasic release of FD from reservoirs R_0_ and R_2_. The two phases are symbolically designated by the lines α and β.

Figure 3a shows the release kinetics of FD from different radial positions after the immersion of the capsules in the PBS dissolution medium. Diffusion of the drug inside the gelatin matrix and its release from the surface of the capsules are triggered by the penetration of the solvent into the drug reservoir. The experiments were performed with capsules containing a single reservoir.

The release from the reservoir R_0_, which is formed on the inner surface of the capsule, starts immediately after the capsule immersion in the dissolution medium. The release from this reservoir is almost complete within approximately 30 min of dissolution, before the eventual closing of the inner cavity due to the swelling of the capsule. Reservoirs R_1_ and R_3_ are both separated from the surfaces of the capsule by an average of two layers. Nevertheless, it was systematically observed that the release from R_3_, which is closer to the outer surface of the capsule, is faster than the release from R_1_, which is closer to the inner surface. After 8 h of dissolution, 95% of the FD is released from reservoir R_0_ in comparison to 56% from R_1_, 50% from R_2_ and 72% from R_3_.

Figure 3b shows the biphasic release of FD from reservoirs R_0_ and R_2_. The blue curve represents the release kinetics of FD from two reservoirs within the same capsule; the pink curve denotes the superposition of two release kinetics of FD from two reservoirs, each located within a single capsule. The curves show a perfect layering and they exhibit a biphasic release profile.

### 3.3. Biphasic Bidrug Release

**Figure 4:**
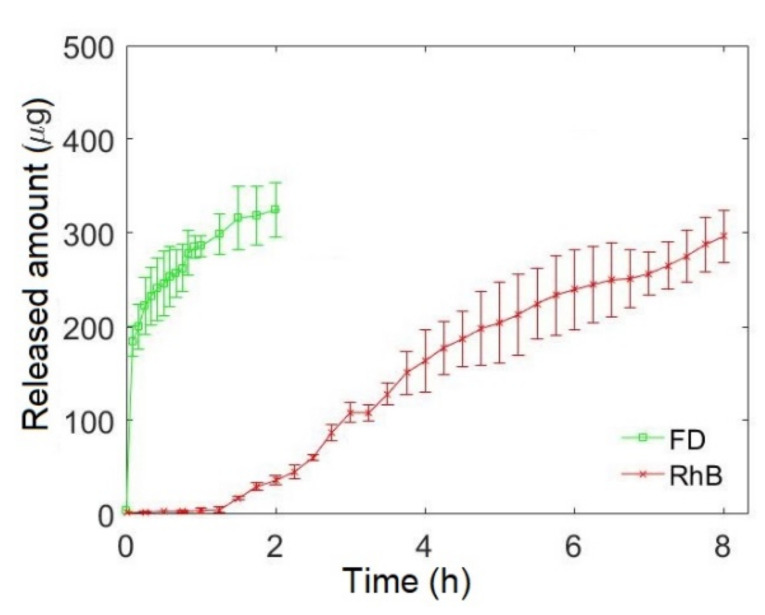
Biphasic bidrug release of FD from R_0_ and RhB from R_2_.

Different drugs can be loaded in the reservoirs. To demonstrate this feature, we loaded reservoirs R_0_ and R_2_ with FD and RhB, respectively. In this study, we used FD and RhB not only because they are good tracers but also because they have different characteristics. FD is hydrophilic and RhB is highly lipophilic. Figure 4 shows the release profile of FD and RhB from reservoirs R_0_ and R_2_, respectively. Approximately 70% of FD loaded to R_0_ was released during the first hour, while no release of RhB occurred from R_2_ during this period. The release of RhB starts with approximately 1.5 h lag time. The release curve of FD was truncated at 2 hours, since the dissolution of RhB, which has a basic nature, might modify the fluorescence intensity of FD due to the change of the portion of energy absorbed by different ionic states and their different quantum yields [23], which gives the false impression of the continuing release of the dye. 

## 4. Discussion

Crosslinked gelatin films were found to be a convenient material for the design of the rolled-up capsules suitable for the programmable drug release. DHT treatment was applied on the gelatinous matrix at 150 °C for 8 h and produced gelatin films characterized by a limited rate of swelling and resistance to dissolution under physiological conditions (Appendix A). The 60-µm-thick films remain sufficiently flexible after the treatment so they can be rolled up over a cylindrical support that is a few millimeters wide without cracking. The rolls produced in the present study correspond approximately to the “000” type oral administration capsules of the French Pharmacopeia [24]. Smaller rolled capsules can be produced as well by reducing the thickness of the films and the diameter of the cylindrical support. We did not use any plasticizer to modify the mechanical properties of gelatin films to ensure the simplicity of the system. The reservoirs were formed by spreading the solutions using a micropipette in the specific limits of the reservoir. This manual approach was sufficiently precise to provide good reproducibility of the kinetics, which was adequate to meet the objectives of the present study. For potential clinical applications, a more automated fabrication protocol using materials inkjet printing [15] would be preferable, but it should be adapted for rapid printing of sufficiently large quantities of the real drugs. 

In view of the limited stability of the rolled capsules in the strongly acidic media, they might be more suitable for biphasic drug release in the small intestine, rather than in the stomach. The rolls will be encased in the gastro-resistant capsules, e.g., on the base of Eudragit^®^L100-55, which is dissolved above pH 5.5 and designed for drug release in the mid to upper small intestine. A good example of such a biphasic release in the small intestine is the release of diclofenac sodium, which belongs to the class of the nonsteroidal anti-inflammatory drugs (NSAIDs) [25]. However, if the stability issues of the rolled up gelatin capsules in the acidic media are resolved in the future, it would be interesting to consider them as a biphasic release gastroretentive dosage form. This application will be favored by the fact that the capsules swell strongly in the highly acidic media. Therefore, the capsules may be designed in such a way that they will be swallowable, but will be retained from passage through the pylorus due to swelling in the gastric juice. 

The release curve of FD from R_0_ showed QR kinetics. In fact, positioning the drug on the inner surface (R_0_) of the capsule assures immediate release of FD. The release from the reservoirs, embedded in the roll, can be classified as sustained, with the release rates depending on the radial position of the reservoirs. The release from the R_1_ reservoir is relatively slow because of the progressive obstruction of the central hole due to the swelling of the gelatin matrix. This process reduces the interface between the inner cavity and the dissolution media, making it difficult to drain and evacuate the drug from the cavity. As expected, the release from reservoir R_2_, which is at the greatest distance from both surfaces of the capsule, is the slowest. A biphasic drug release is achieved in this system from the capsules containing the R_0_ and the R_2_ reservoirs. The system is suitable not only for the biphasic release of a single drug, but also for the chronomodulated release of two (or more) different drugs, as we have demonstrated with the use of the model fluorescent drugs, FD and RhB, loaded in the reservoirs R_0_ and R_2_, respectively. As an example of a biphasic release of two drugs, one can mention a recent study on the immediate release of atenolol, a beta blocker medication used to treat high blood pressure and heart-associated chest pain, and sustained release of lovastatin, a statin medication used for the treatment of high blood cholesterol [26].

The characteristic swelling time of the capsules is of the same order as the lag time of the release from the embedded reservoirs (see Figure 2 and Figure 3), therefore the release should be classified as diffusion-controlled and swelling-controlled. This greatly complicates the theoretical description of the release kinetics. Nevertheless, the sustained-release phase of the biphasic release (Figure 3b) can be classified as pseudo-zero order one. 

## 5. Conclusions

We have designed rolled-up capsules for a programmable biphasic drug release. The biphasic kinetics were achieved due to specific geometry of the capsules, rather than due to the different matrix disintegration rates, which is the method used in traditional bilayer tablet biphasic release systems. The capsules were formed by rolling up thermally crosslinked gelatin strips, on the surface of which different reservoirs were distributed and loaded with FD as the model drug. The capsules were maintained in the rolled state using transglutaminase. Quick release was effectuated from the inner surface of a cylinder-like capsule during the first minutes of immersion in the dissolution media. Sustained release was achieved via embedding a drug reservoir between the layers of the rolls. Moreover, the design of the capsule was suitable for the dual-drug release in a chronomodulated manner, as demonstrated by the experiments with capsules containing FD and RhB in the QR and SR reservoirs, respectively. This concept, which is based on a single polymer, can be implemented on a large scale. As the next step, the system will be explored in vitro with the use of the real drugs, such as diclofenac sodium, for the biphasic release in the Fasted State Simulated Intestinal Fluid (FaSSIF).

## Figures and Tables

**Table 1:** The lateral coordinates of the reservoirs before rolling.

Reservoir	Turn	x_1_ [mm]	x_2_ [mm]
R_0_	1	0	17
R_1_	3	35	53
R_2_	6	91	110
R_3_	8.5	140	160

**Figure A1:**
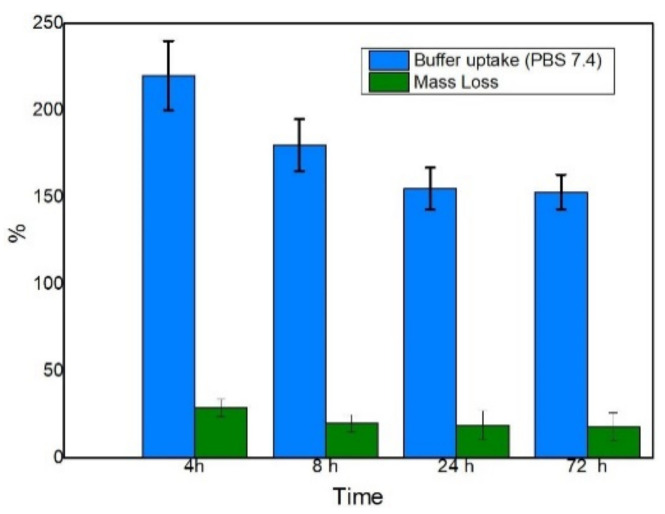
Swelling capacity and mass loss % of gelatin films after immersion in PBS (pH = 7.4).

## Data Availability

Not applicable.

## Appendix A

The results show that the swelling capacity seems to be strongly dependent on the heating time. Aiming to understand the behavior of the films during the first hours of immersion, the swelling capacity of the films was studied and plotted against time (Figure A1). The results show mass variations, suggesting that the swelling phenomenon interferes with the polymer mass loss. Indeed, these systems may exhibit not only swelling-deswelling behavior but also polymer degradation. The swelling capacity of the untreated films increases substantially during the first 10 min of immersion in physiological conditions, then they dissolve. The films treated for 1 h swelled up to 850% and turned into gel and could not be sustained for more than 2 h in PBS. In contrast, the films treated for 4 h, 8 h, 24 h and 72 h showed a swelling percentage equal to 220 ± 20%, 180 ± 15%, 155 ± 15% and 153 ± 13%, respectively, after 24 h of immersion in PBS. However, mass loss becomes almost constant after 8 h of heat treatment. Therefore, the films treated during the first 8 h were retained and used as the matrix to design the rolled-up capsules.

## Appendix B

The extent of crosslinking was determined by measuring the amount of free or unreacted amino groups in the gelatin films [17]. The following protocol was adapted from a procedure described by Kale and Bajaj [18]. First, 25 µg of thermally crosslinked gelatin film was placed in a 100 mL screw cap test tube. Then, 2 mL of 4% NaHCO_3_ (Sigma–Aldrich) and 2 mL of 0.5% 2,4,6-Trinitrobenzensulphonic acid (TNBS) (Sigma–Aldrich, Taufkirchen, Germany) were added to the tube. The mixture was heated to 40 °C for 5 h, then 6 mL of 6 N HCL was added. The mixture was autoclaved at 120 °C for 1 h in an oven for total hydrolyzation, diluted to 20 mL with distilled water, then extracted with two 40 mL portions of ether to remove the excess of the unreacted TNBS. To evaporate the residual ether, the mixture was heated for 10 min in a hot water bath. The absorbance was read at 346 nm using a Jasco double-beam spectrophotometer against a reagent blank prepared with same procedure. The number of ε-amino groups, C, expressed in [*moles*]/[*grams of gelatin*] units was calculated as the average of three measurements using the following equation:

(A1) $$C=\frac{2\times Abs\times V}{1.46\times 10^{-4}\times b\times m_{gel}}\;$$

where Abs is the absorbance, $1.46\;L\cdot \mathrm{mol}e^{-1}\cdot cm^{-1}$ is the molar absorptivity of TNP-lys, b=1 cm is the cell path length, $m_{gel}$ is the sample weight in grams, and V= 0.02L is the volume of the water solution containing hydrolyzed protein after the ether evaporation. The ε-amino content of the uncrosslinked gelatin film was estimated to be $29\times 10^{-5}$ moles per gram of gelatin. The average molecular mass of an amino acid is usually taken to be equal to 110 Da. Therefore, the ε-amino content can be estimated to be 32 ε-amino groups per gelatin molecule of 1000 amino acid residues.

The extent of reaction was defined as:

(A2) $$f=1-\frac{C_{cr}}{C_{0}}\;$$

where $C_{cr}$,$C_{0}\;\mathrm{are}\;\mathrm{the}\;$mean amount of moles of the ε-amino groups per gram of gelatin in the crosslinked and uncrosslinked films, respectively. From the definition, it follows that f→0 if $C_{cr}\to C_{0}$ and f→1 if $C_{cr}\to 0$. From (1), it also follows that:

(A3) $$f=1-\frac{Abs_{cr\;}}{Abs_{0}}$$

where $A\;bs_{cr}$, $Abs_{0\;}$are the absorptions of the crosslinked and uncrosslinked gelatin. The extent of the reaction was found to be *f* = 0.36; that is, approximately 36% of the ε-amino groups, or 13 per molecule of 1000 amino residues, was consumed in the crosslinking reaction.

## Appendix C

The shape of the roll can be well approximated, in the polar coordinates, by the Archimedean spiral:

(A4) $$r=r_{0}+\frac{h}{2\pi }\varphi$$

where r is the radius-vector, φ is the angle of rotaion of the vector, h is the thickness of the film. Suppose that a reservoir should be placed on the nth turn of the roll. Let $x_{1}$, $x_{2}$ be the coordinates of the limits of the reservoir along the length of the stripe before rolling. These limits can be found as:

(A5) $$x_{1}=\overset{2\pi \left(n-1\right)}{\underset{0}{\int }}\frac{dl}{d\varphi }d\varphi =\overset{2\pi \left(n-1\right)}{\underset{0}{\int }}\sqrt{{\left(r_{0}+\frac{h}{2\pi }\varphi \right)}^{2}+{\left(\frac{h}{2\pi }\right)}^{2}}d\varphi$$

(A6) $$x_{2}=\overset{2\pi n}{\underset{0}{\int }}\frac{dl}{d\varphi }d\varphi =\overset{2\pi n}{\underset{0}{\int }}\sqrt{{\left(r_{0}+\frac{h}{2\pi }\varphi \right)}^{2}+{\left(\frac{h}{2\pi }\right)}^{2}}d\varphi$$

where dl is the element of the length of the spiral corresponding to the rotation of the radius-vector by dφ. The limits of the reservoirs $R_{0}$, $R_{1}$, $R_{2}$ and $R_{3}$, calculated by the formulas (C2), (C3) for the experimental values h=0.06 mm, $r_{0}=2.75\;\mathrm{mm}$ are given in Table 1 of the main text.
